# Analysis of Strength and Homogeneity of Different Concrete Specimens Prepared Under a High-Frequency and Low-Power Piezoelectric Excitation System

**DOI:** 10.3390/ma19081600

**Published:** 2026-04-16

**Authors:** Nabi İbadov, Gürcan Çetin, Ercüment Güvenç, Murat Çevikbaş, İsmail Serkan Üncü, Kamil Furkan İlhan

**Affiliations:** 1 Faculty of Civil Engineering, Department of Production Engineering and Management in Construction, Warsaw University of Technology, Al. Armii Ludowej 16, 00-637 Warszawa, Poland; nabi.ibadov@pw.edu.pl; 2 Information Systems Engineering, Faculty of Technology, Muğla Sıtkı Koçman University, 48000 Muğla, Türkiye; gcetin@mu.edu.tr; 3 Rectorate Units, Department of Informatics, Muğla Sıtkı Koçman University, 48000 Muğla, Türkiye; eguvenc@mu.edu.tr; 4 Department of Civil Engineering, Faculty of Technology, Isparta University of Applied Sciences, 32040 Isparta, Türkiye; muratcevikbas@isparta.edu.tr; 5 Department of Electrical and Electronics Engineering, Faculty of Technology, Isparta University of Applied Sciences, 32040 Isparta, Türkiye; furkanilhan@isparta.edu.tr

**Keywords:** concrete, compressive strength, ultrasonic pulse velocity, piezoelectric vibration, surface roughness, image processing

## Abstract

Ensuring the durability and safety of modern infrastructure critically depends on the quality and strength of concrete. The Ultrasonic Pulse Velocity (UPV) method is a widely used non-destructive testing technique for evaluating concrete properties; however, factors such as aggregate size distribution, compaction methods, and surface quality can significantly influence UPV results and their correlation with compressive strength. This study investigates the effects of different aggregate sizes and an innovative vibration-assisted compaction method—developed using piezoelectric (PZT) transducers—on the mechanical, ultrasonic, and surface properties of concrete. Four distinct aggregate size distributions were employed to produce sixteen concrete specimens with constant mix proportions. Unlike conventional low-frequency, high-power vibration practices, a high-frequency (40 kHz), low-power (120 W) vibration protocol was applied through PZT elements placed within the molds to enhance compaction and reduce entrapped air. Experimental results indicated that the heaviest specimen (7.13 kg) was the medium-aggregate sample compacted using tamping and rodding methods. The highest UPV value (4143 m/s) was obtained from the coarse-aggregate specimen subjected to three minutes of vibration. In contrast, the best compressive strength performance (22.73 MPa) was observed in the medium-aggregate specimen without any vibration treatment. The findings revealed that both aggregate size and advanced vibration techniques have significant effects on the mechanical properties, ultrasonic response, and surface quality of concrete. In addition, a proof-of-concept portable surface-finishing prototype consisting of a steel plate instrumented with multiple PZT transducers was developed, and preliminary trials qualitatively suggested improved surface leveling when applied in contact with the concrete surface. Surface roughness was quantified via image processing (Light Map 150 and Specular Map 150). The rough-area fraction decreased from ~29.8% in the untreated specimen to ~4.3% after ultrasonic application, indicating a marked improvement in surface leveling and overall surface quality. The results indicate that the applied PZT vibration protocol did not improve compressive strength; in several cases, particularly under prolonged excitation, a reduction in strength was observed. In contrast, a significant improvement in surface quality was achieved, with the rough-area fraction decreasing from approximately 29.8% to 4.3%. However, due to the limited number of specimens, the findings should be interpreted as preliminary. Overall, the method appears more promising as a surface enhancement technique rather than a direct alternative to conventional compaction methods.

## 1. Introduction

Concrete is one of the most widely used construction materials worldwide due to its durability, cost-effectiveness, and versatility of application. With an annual production exceeding 10 billion cubic meters, concrete is the second most consumed material globally after water. Among its mechanical properties, compressive strength is primarily governed by its constituents, including cement, water, chemical and mineral admixtures, and, most importantly, aggregates. The structural characteristics of aggregates—such as particle size distribution, density, and surface properties—significantly influence the internal structure and strength of concrete [1,2].

Recent structural failures observed after the 2023 Kahramanmaraş earthquakes in Türkiye have once again highlighted the critical importance of accurately evaluating and improving concrete performance, particularly compressive strength and internal integrity in seismic regions. These events have emphasized the need for reliable construction practices and improved material characterization techniques.

Proper compaction during casting plays a crucial role in determining the mechanical performance and durability of concrete. Conventional compaction methods typically rely on low-frequency, high-power mechanical vibration. These techniques are widely adopted in practice due to their effectiveness in reducing entrapped air and improving density. However, under certain conditions—such as complex formworks or varying aggregate distributions—conventional vibration may lead to non-uniform compaction, segregation, or insufficient removal of air voids, which can adversely affect the mechanical performance and durability of concrete.

In recent years, alternative compaction approaches based on high-frequency excitation have attracted increasing attention. Among these, piezoelectric transducer (PZT)-based systems offer a novel method by introducing controlled high-frequency oscillations during the casting process. Unlike conventional low-frequency vibration, high-frequency excitation may influence the distribution of particles at a finer scale, potentially affecting both surface characteristics and internal structure. However, the overall effectiveness of such systems in improving concrete performance remains unclear and requires systematic investigation.

Previous studies have extensively investigated the effects of vibration-based compaction on concrete properties, particularly compressive strength and durability. In addition, numerous works have explored the relationship between Ultrasonic Pulse Velocity (UPV) and mechanical performance as a non-destructive evaluation method. More recently, image processing techniques have been applied to assess surface quality and detect defects in cementitious materials [3,4,5].

Several researchers have examined the influence of ultrasonic and high-frequency vibration on concrete properties. Popovics and Popovics [4] applied vibration at 54 kHz and 120 kHz and reported that UPV remained largely constant up to a certain loading level. Gudra and Stawiski [5] investigated high-frequency excitation and observed that compaction near the excitation source could be non-uniform. Ohdaira and Masuzawa [6] reported that ultrasonic velocity decreased with decreasing water content. Del Rio et al. [7] suggested that vibration-based approaches can be used to estimate the hardening behavior of concrete. Abo-Qudais [8] reported that UPV increased with curing time, while Juradin et al. [9] demonstrated that both insufficient and excessive vibration negatively affect concrete quality. Bompan and Haach [10] highlighted that early-age microcracking may significantly influence UPV measurements. Zhang et al. [11] and Dengaev et al. [12] reported that high-frequency vibration may improve compressive strength under certain conditions, whereas Xiong et al. [13] showed that vibration efficiency strongly depends on power and duration. Additional studies have emphasized that excessive vibration may lead to segregation and reduced strength despite improvements in surface quality [14,15,16,17,18,19].

Recent studies have also focused on the integration of advanced image processing and digital analysis techniques to better characterize surface defects and heterogeneity in cementitious materials. Machine vision-based approaches have enabled quantitative evaluation of surface roughness and defect distribution, improving the objectivity of surface quality assessments [20,21,22]. Furthermore, recent research indicates that high-frequency excitation may alter particle packing behavior and microstructural development in fresh concrete, although its influence on compressive strength remains inconsistent across different experimental conditions [23,24].

In addition, several studies have highlighted that non-destructive testing methods such as UPV are highly sensitive to internal defects, density variations, and microcracking, but may not always directly correlate with compressive strength, particularly in heterogeneous or non-uniform systems [25,26,27]. These findings suggest that a multi-parameter evaluation approach is necessary to fully understand the effects of unconventional compaction techniques.

Recent advancements have also explored hybrid evaluation approaches combining ultrasonic testing with digital imaging to improve the reliability of concrete quality assessment. These studies demonstrate that integrating multiple techniques provides a more comprehensive understanding of internal defects and surface conditions, particularly in heterogeneous materials [28,29,30]. Moreover, investigations into ultrasonic and high-frequency excitation systems indicate that vibration parameters such as frequency, duration, and power significantly influence microstructure development and mechanical performance. While improvements in density and surface quality are often reported, excessive or prolonged excitation may induce microcracking and strength reduction [8,31].

Despite these advancements, the combined effects of high-frequency, low-power excitation on mechanical properties, ultrasonic response, and surface characteristics have not been sufficiently investigated, especially in relation to different aggregate size fractions. This highlights a clear gap in the current literature.

Assessing the compressive strength of concrete is essential for ensuring structural safety. Both destructive and non-destructive methods are widely used for this purpose. While destructive tests provide direct measurement of load-bearing capacity, the Ultrasonic Pulse Velocity (UPV) test evaluates wave propagation through the material and provides insight into internal homogeneity and defects [2]. However, the relationship between UPV and mechanical strength remains complex, particularly under unconventional excitation conditions.

Another critical factor affecting concrete performance is the presence of entrapped air during casting. Reducing these air voids improves density and strength. While conventional vibration techniques are commonly used for this purpose, recent studies have explored alternative vibration strategies to enhance compaction efficiency and surface quality.

In this context, the present study investigates the effects of high-frequency (40 kHz), low-power piezoelectric transducer (PZT) excitation on concrete specimens prepared with different aggregate size fractions. The novelty of this work lies in the combined evaluation of compressive strength, Ultrasonic Pulse Velocity (UPV), surface quality, and internal homogeneity within a single experimental framework.

Furthermore, a quantitative image processing approach is employed to assess surface roughness, providing a measurable indicator of surface quality improvement. Unlike conventional studies that primarily focus on mechanical performance, this study highlights the presence of competing effects under high-frequency excitation conditions, offering a more balanced and realistic evaluation of the method. The findings are expected to provide guidance for optimizing high-frequency vibration parameters in future studies.

## 2. Materials and Methods

### 2.1. Concrete Mix Design and Specimen Preparation

This study is designed as a proof-of-concept experimental investigation aimed at evaluating the feasibility and qualitative effects of high-frequency, low-power piezoelectric excitation on concrete properties.

A total of sixteen concrete specimens with identical mix proportions were prepared using four different aggregate size fractions at a water-to-cement ratio of 0.5. The aggregate fractions comprised fine (0–4 mm), medium (4–8 mm), medium-coarse (8–16 mm), and coarse (16–22 mm) aggregates. The mixture proportions by mass were determined based on standard concrete formulations, and for each specimen, 1.25 kg of sand, 1.25 kg of water, 2.5 kg of cement, and 2.7 kg of aggregate were used. The mixtures were cast into standard plastic cube molds (15 × 15 × 15 cm) as well as custom-designed metal molds of the same dimensions that were suitable for vibration application. Prior to casting, the mold surfaces were oiled to facilitate demolding of the hardened specimens and to prevent surface defects. In this study, CEM I 42.5 R cement produced by Göltaş Cement Inc. (Isparta, Türkiye) was used as the binder in all mixtures. Aggregates were sourced locally and classified based on standard sieve analysis. The aggregates were obtained from local sources and classified according to standard sieve analysis. Tap water was used for mixing. No chemical admixtures were incorporated into the mixtures.

Due to the limited number of specimens tested for each condition, the results should be interpreted as preliminary and indicative rather than statistically conclusive. The experimental design is therefore intended to provide initial insights and proof-of-concept observations rather than statistically generalized conclusions.

**Figure 1:**
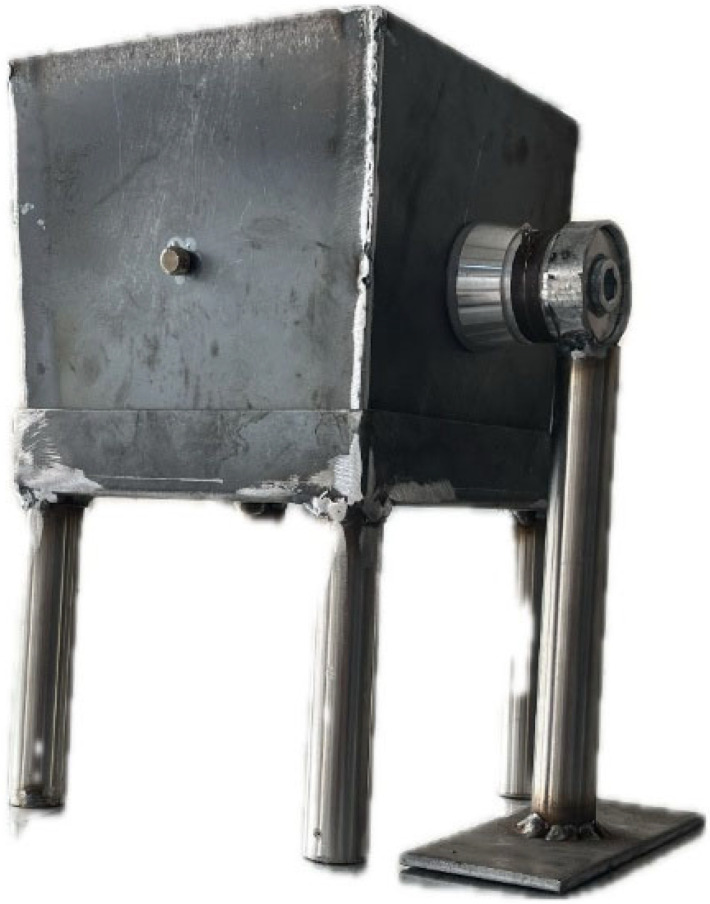
Mold system designed for vibration application.

A piezoelectric transducer was positioned at the midpoint of the lateral surface area of the metal mold. In addition, two transducers were installed on the base of the mold, placed at locations equidistant from the center points. The transducers were secured to bolts that had been welded onto the metal surface. To prevent detachment from the surface and to ensure stable positioning under vibration, dedicated transducer mounting supports were designed. The complete setup of the custom-designed metal molds used for the vibration-treated specimens is shown in Figure 1.

### 2.2. Compaction Methods

**Figure 2:**
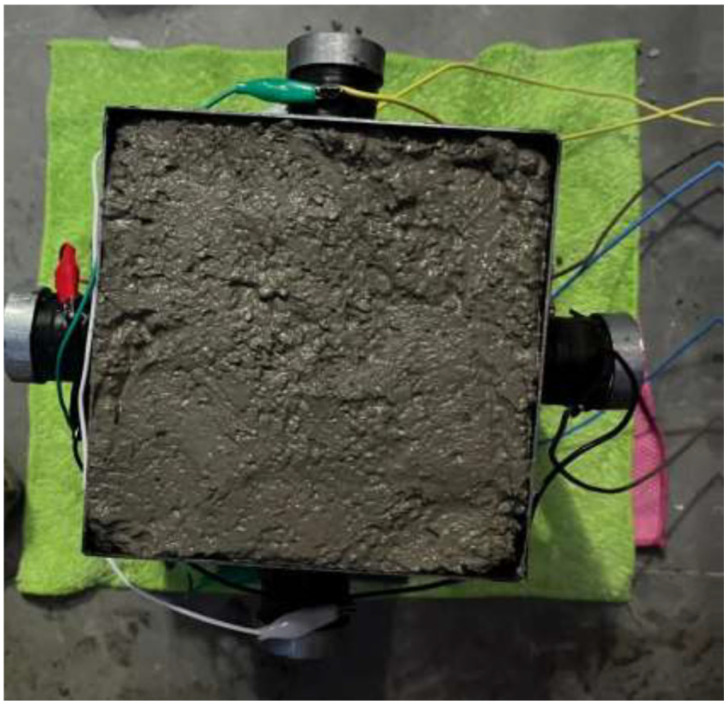
Concrete mixture subjected to 40 kHz vibration.

For each aggregate fraction, one specimen was left untreated and used as a reference. A second specimen was manually compacted by rodding and tamping using a steel rod and a tamper. The remaining two specimens were subjected to PZT-based vibration-assisted compaction. Vibration was applied at 40 kHz with a driver power of 120 W for durations of 1 min and 3 min, respectively. The transducers were mounted on the base and lateral faces of the mold and were activated sequentially from the bottom upward. This approach was intended to promote effective removal of entrapped air and to provide a more homogeneous compaction process progressing from the base toward the top. An example concrete specimen subjected to vibration is shown in Figure 2.

The excitation frequency of 40 kHz was selected based on the operating resonance of the piezoelectric transducer system and represents a practical engineering parameter rather than an optimized value.

The selected excitation durations (1 and 3 min) were chosen to compare moderate and prolonged exposure conditions during the casting process. The experimental procedure steps are presented below.

The experimental procedure consists of the following steps:

1. Preparation of concrete mixtures;
2. Specimen casting;
3. Application of PZT excitation;
4. Curing process;
5. Compressive strength testing;
6. UPV measurements;
7. Surface imaging;
8. Image processing and analysis.

**Figure 3:**
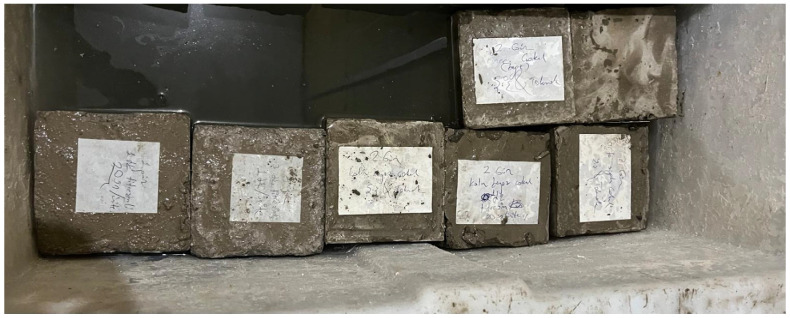
Curing of the specimens in a water-curing tank for hydration/strength development.

In total, four specimens were prepared for each aggregate fraction—untreated, rodded and tamped, vibrated for 1 min, and vibrated for 3 min—and were cured in a water-curing tank for 28 days to allow proper hydration and strength development. The specimens stored in the curing tank are shown in Figure 3.

### 2.3. Test Procedures

After demolding, the specimens were kept in a water-curing tank for 28 days to ensure consistent hydration and strength development. Following the curing period, three tests were performed. The UPV test was conducted to measure the ultrasonic wave propagation velocity within hardened concrete. Compressive strength was determined using a standard compression testing machine to identify the maximum load-bearing capacity of the specimens. Specimen mass was measured using a precision balance to evaluate density-related differences associated with the different compaction methods.

### 2.4. Piezoelectric Vibration System

**Figure 4:**
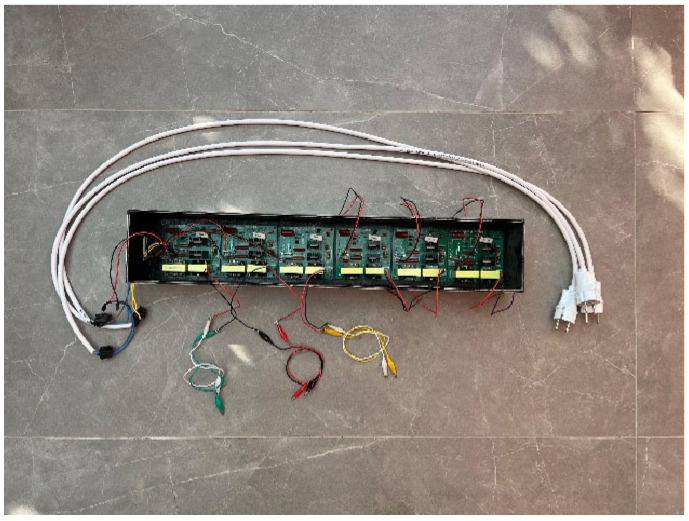
Protective enclosure for the piezoelectric transducer driver.

The vibration system was established using bolt-clamped piezoelectric transducers with a resonance frequency of 40 kHz and a rated power of 60 W per transducer. The transducers were driven by a custom-designed 120 W ultrasonic piezoelectric driver based on a half-bridge inverter topology. The system was configured to apply controlled high-frequency excitation during the initial setting stage of fresh concrete. Transducer mounting and mold modifications were carefully engineered to ensure consistent and effective vibration transmission. The driver enclosure, including the electrical connections, is shown in Figure 4.

## 3. Results

### 3.1. General Evaluation of the Experimental Results

Within the experimental program, three key parameters of the hardened concrete specimens were evaluated: mass, Ultrasonic Pulse Velocity (UPV), and compressive strength. A total of sixteen specimens prepared with different aggregate size fractions and various compaction techniques were tested. Detailed results are presented in Table 1, Table 2 and Table 3.

The UPV measurements summarized in Table 1 showed substantial differences across aggregate fractions and compaction methods. Specimens containing coarse aggregate exhibited the highest UPV values. The coarse-aggregate specimen subjected to 3 min of vibration achieved the maximum UPV of 4143 m/s. However, no clear linear relationship was observed between UPV and compressive strength. This finding is consistent with previous reports in the literature indicating that UPV alone may not reliably predict mechanical performance.

The specimen masses reported in Table 2 followed the expected trends: specimens produced with medium and coarse aggregate were generally heavier. Manual rodding and tamping resulted in the densest specimens, whereas longer vibration durations tended to reduce specimen mass. This trend may be attributed to more effective removal of entrapped air, together with the potential emergence of segregation effects under excessive vibration durations.

These findings indicate that the applied high-frequency vibration does not enhance compressive strength under the tested conditions and may even have a detrimental effect when applied for longer durations.

This further supports the argument that density-related parameters play a more dominant role in strength development compared to UPV under high-frequency excitation conditions.

The compressive strength results (Table 3) indicated that, in general, the untreated specimens exhibited higher strength than the vibration-treated specimens. This trend became more pronounced for longer vibration durations. The highest compressive strength was obtained for the untreated medium-aggregate specimen (22.73 MPa). The strength reduction observed under prolonged vibration (3 min) is attributed to possible microstructural damage and/or segregation effects induced by excessive vibration energy. Overall, the maximum UPV was recorded for the coarse-aggregate specimen exposed to 3 min of piezoelectric vibration (4143 m/s). The heaviest specimen (7.13 kg) was the medium-aggregate specimen compacted by rodding and tamping.

### 3.2. Quantitative Analysis of the Obtained Data

**Figure 5:**
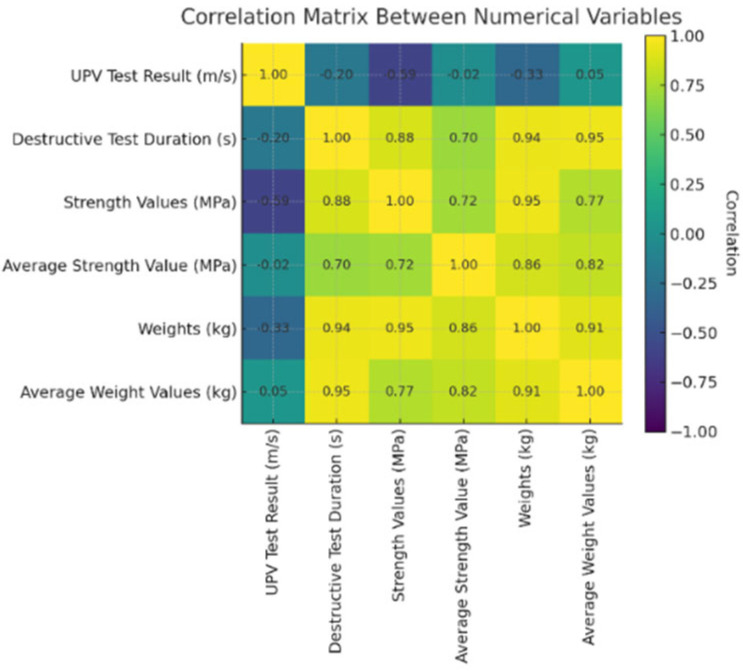
Correlation matrix among the numerical variables.

The correlation matrix of the obtained numerical data is presented in Figure 5. Based on the correlation analysis, a moderate positive correlation (r ≈ 0.63) was found between specimen mass and compressive strength. In contrast, a weak positive correlation (r ≈ 0.23) was observed between UPV and compressive strength. In addition, a strong positive correlation (r ≈ 0.80) was obtained between the average specimen mass and the average compressive strength. Furthermore, the correlation between failure time and compressive strength was very weak (r ≈ 0.11). Overall, these results suggest that mass is a meaningful indicator of compressive strength, whereas UPV alone is not a sufficient predictor of strength.

To improve the reliability of the findings, multiple evaluation methods, including mechanical testing, UPV measurements, and image-based surface analysis, were employed.

**Figure 6:**
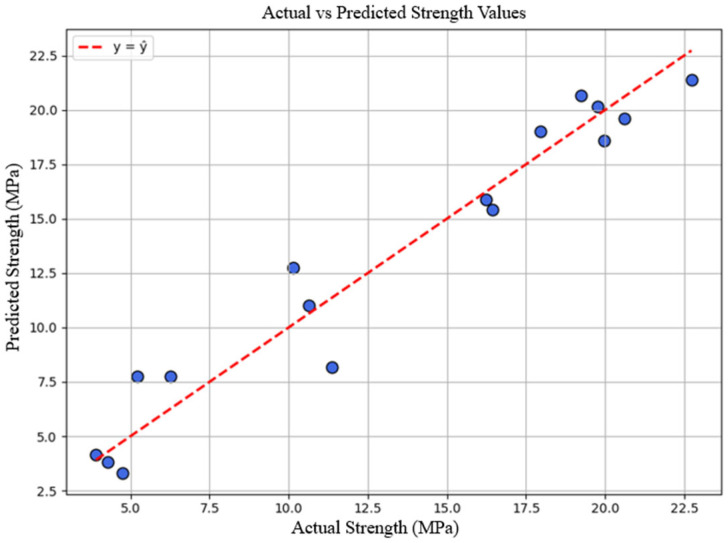
Regression model for predicting compressive strength.

A multiple linear regression model was developed using UPV and specimen mass as predictor variables. The resulting model exhibited strong predictive performance, with a high coefficient of determination (R^2^ = 0.945). In this model, specimen mass provided a meaningful contribution to strength prediction (≈20.8 MPa per 1 kg increase), whereas UPV showed a limited contribution (≈0.0807 MPa per 1 m/s increase). This indicates that, under the tested conditions, specimen density-related effects played a dominant role in strength development. The regression model for predicting concrete strength is presented in Figure 6.

### 3.3. Examination of Relationships Among the Data

**Figure 7:**
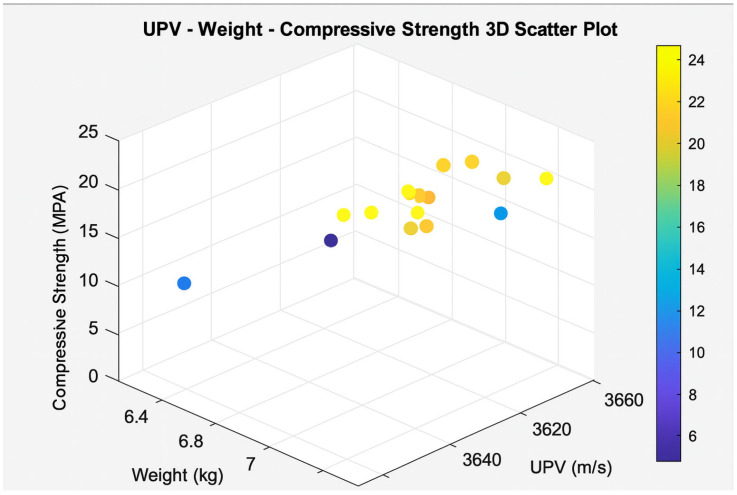
Relationship among UPV, mass, and compressive strength.

**Figure 8:**
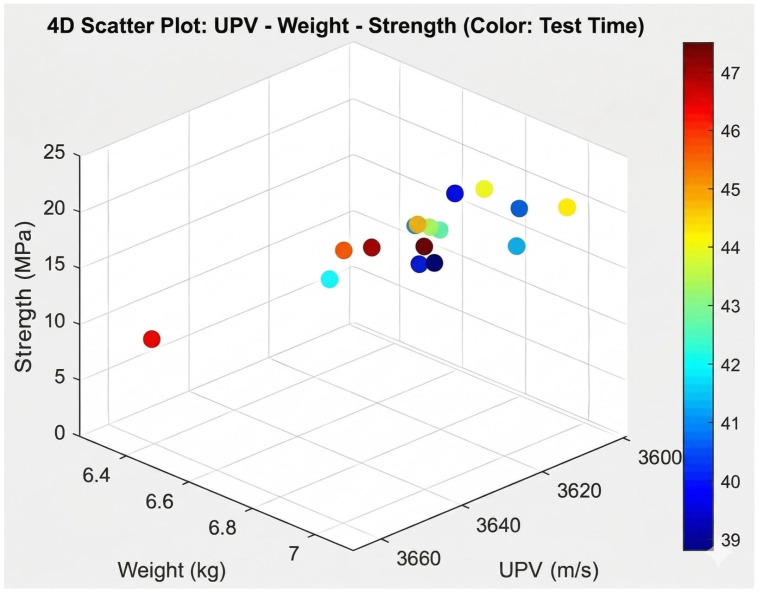
Relationship among UPV, mass, and compressive strength with respect to test duration.

**Figure 9:**
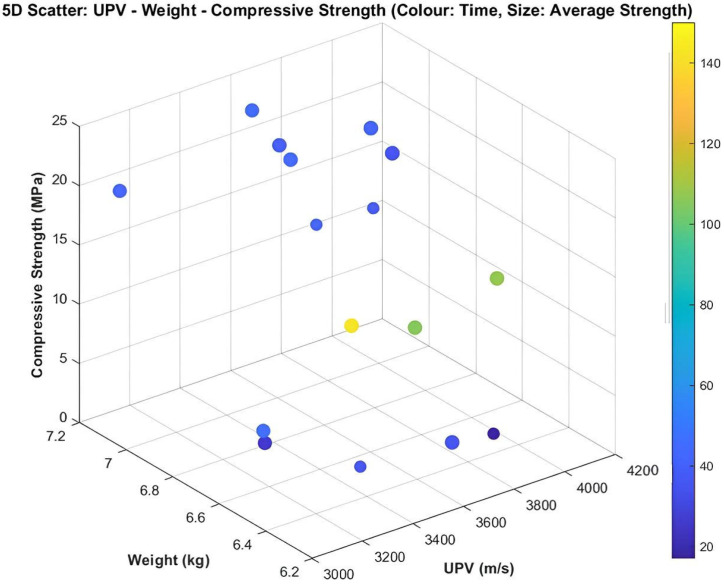
Relationship of UPV, mass, compressive strength, and test duration to the average strength value.

To investigate the complex interactions among the parameters, advanced visualization methods were employed. Accordingly, 3D, 4D, and 5D plots were generated from the experimental dataset. The 3D plots indicated that heavier specimens generally exhibited higher compressive strength, whereas UPV trends were not consistent. In the 4D plots, where failure time was incorporated in addition to the variables used in the 3D plots, higher-strength specimens were observed to fail more rapidly under the test conditions. The 5D plot further extended the 4D representation by including the average strength value, thereby highlighting the importance of specimen density and aggregate fraction on mechanical performance and revealing the complex role of vibration duration. The corresponding 3D, 4D, and 5D visualizations are presented in Figure 7, Figure 8 and Figure 9, respectively.

**Figure 10:**
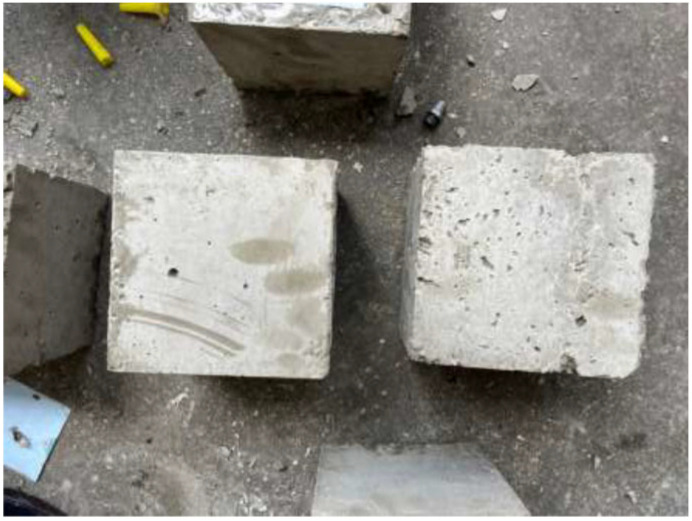
Volumetric surface effect of PZT application on specimens with a constant mix composition.

**Figure 11:**
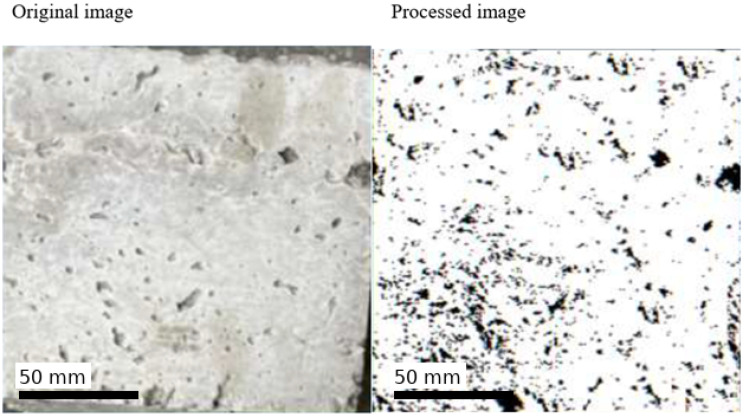
Morphological analysis of the untreated surface obtained via image processing.

**Figure 12:**
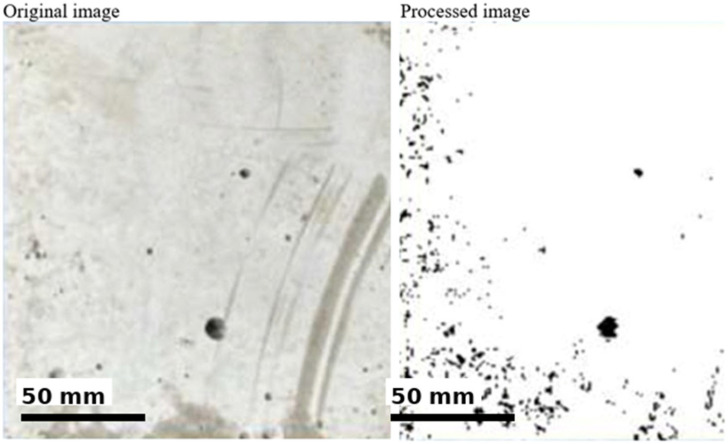
Image processing-based morphological representation of the actuated surface.

In this context, surface roughness was evaluated by analyzing the luminance distribution and specular reflection components of the images on a pixel-wise basis, using image processing algorithms developed by the authors and referred to as Light Map 150 and Specular Map 150. Both methods are widely used for the visual yet quantitative evaluation of surface morphology. The analyses indicated that approximately 29.8% of the surface area of the untreated specimen was classified as rough. After applying the ultrasonic system, evaluation using the same image processing techniques showed that the rough-area fraction decreased to 4.3%. This reduction suggests a pronounced leveling of the outer surface of the hardened concrete. Overall, these results demonstrate that the ultrasonic application has a beneficial effect on surface quality and that this effect can be clearly monitored via image processing methods. The surface roughness levels of the specimens with and without vibration are shown in Figure 10. The original and processed images of the untreated specimen with a rough surface are presented in Figure 11. The original and processed images of the specimen exposed to PZT actuation, exhibiting a lower roughness level, are provided in Figure 12. In both sets of images, rough regions and voids appear as black areas.

Surface images were converted into grayscale and processed using a fixed thresholding approach. A threshold value of 110 (on a 0–255 grayscale scale) was selected based on preliminary calibration tests to distinguish rough regions from smoother areas. Pixels with grayscale values below this threshold were classified as void/rough areas, while higher values represented smoother regions. This fixed thresholding approach ensures reproducibility of the image processing methodology. The threshold value (110) was determined through preliminary calibration tests to ensure consistent separation between rough and smooth regions across all samples.

**Figure 13:**
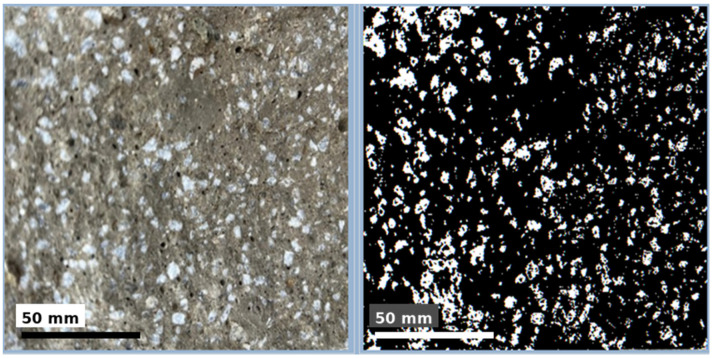
Morphological image obtained via image processing techniques for homogeneity analysis of the concrete internal structure.

To examine how vibration applied to the concrete specimens via piezoelectric transducers influenced internal homogeneity, the concrete matrix was analyzed using the Specular Map image processing technique. Each specimen was split into two halves along its midsection and subsequently processed. The analysis indicated that the high-frequency excitation induced wave patterns that disrupted the expected internal homogeneity of the concrete. Along the direction of transducer contact, both transverse and longitudinal wave components were inferred to promote microcrack formation. In this context, as the vibration duration increased, the homogeneity balance of the mixture deteriorated markedly, depending on the aggregate fraction used. Based on the obtained results, among the four aggregate fractions investigated in this study, the coarse-aggregate specimens exhibited the highest compatibility with the applied 40 kHz excitation in terms of compressive strength performance. As the aggregate size decreased, homogeneity degraded more rapidly, which directly affected compressive strength. The image processing workflow and representative internal-structure results are shown in Figure 13: the left panel depicts the original image of the internal structure, whereas the right panel presents the processed image. In the processed image, white regions indicate the locations of aggregates within the mixture, while black regions represent the remaining constituents of the matrix.

**Figure 14:**
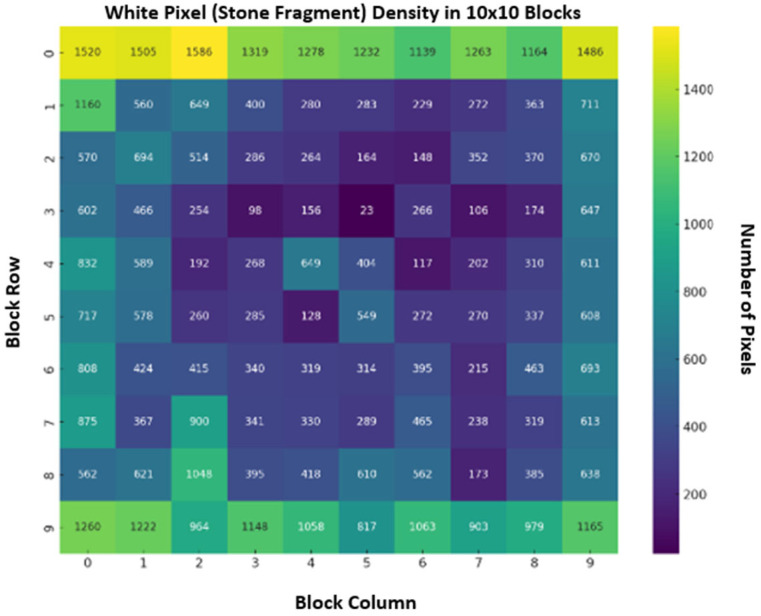
White-pixel density map for homogeneity analysis.

In addition to the processed image, the densities of the black and white pixels were quantitatively compared. The resulting pixel-density map is presented in Figure 14. The map was partitioned into 10 × 10 blocks, and the numerical values within each block represent the white-pixel density in the processed image. As seen in the map, the density levels in the top and bottom rows of blocks are notably high, generally on the order of +1000. In contrast, the substantially lower values observed in several central blocks indicate that the mid-region of the specimen contains little to no aggregate. This pattern suggests that waves induced by the high-frequency excitation altered the spatial distribution of aggregates, causing them to concentrate in specific regions. Accordingly, although the aggregates may appear orderly in the image, the analysis clearly demonstrates an irregular (non-homogeneous) distribution.

This further supports the argument that density-related parameters play a more dominant role in strength development compared to UPV under high-frequency excitation conditions, highlighting the limitations of relying solely on ultrasonic measurements.

## 4. Discussion and Conclusions

This study evaluated the effects of an innovative high-frequency 40 kHz PZT technique on the mechanical and surface properties of concrete specimens prepared with four different aggregate size fractions. The results demonstrate that the proposed method provides selective benefits, particularly in improving surface quality, rather than a direct alternative to conventional compaction. Particularly in terms of mitigating surface roughness. Surface roughness decreased substantially from 29.8% to 4.3%, confirming the potential of the PZT vibration technique to improve surface quality. Specimens compacted by manual rodding and tamping generally exhibited higher density and better mechanical performance than those subjected to prolonged PZT treatment. Prolonged PZT excitation (3 min) reduced compressive strength, particularly in matrix regions with lower aggregate content, likely due to microcrack formation. One of the key findings of this study is that high-frequency piezoelectric vibration can reduce compressive strength in long-term applications. A moderate excitation duration and an optimized frequency may enhance surface quality and compaction without adversely affecting compressive strength. The regression analysis indicated that specimen mass is a strong indicator of compressive strength, whereas UPV exhibits only a weak correlation with strength.

Among non-destructive testing methods, UPV—widely used in practice—produced the most favorable results for specimens with coarse aggregate. However, contrary to expectations, UPV measurements obtained from the medium-coarse aggregate group (one fraction below the largest size) were lower than anticipated. In this context, after the coarse-aggregate group, the next-highest UPV results were observed for the fine-aggregate group with the smallest particle size. It is evident that the applied 40 kHz excitation did not produce a linear effect on compressive strength, as it may have disrupted homogeneity by inducing displacement of aggregates whose size increases proportionally across the fractions. This observation supports the widely reported view that “UPV, when considered alone, does not fully represent concrete strength.” Therefore, UPV data yield more meaningful interpretations when evaluated in conjunction with other parameters.

**Figure 15:**
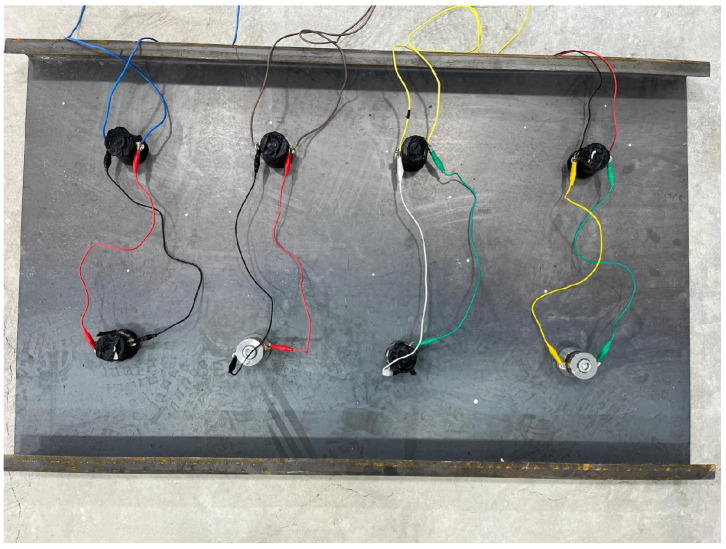
Application plate assembly used to reduce surface roughness.

**Figure 16:**
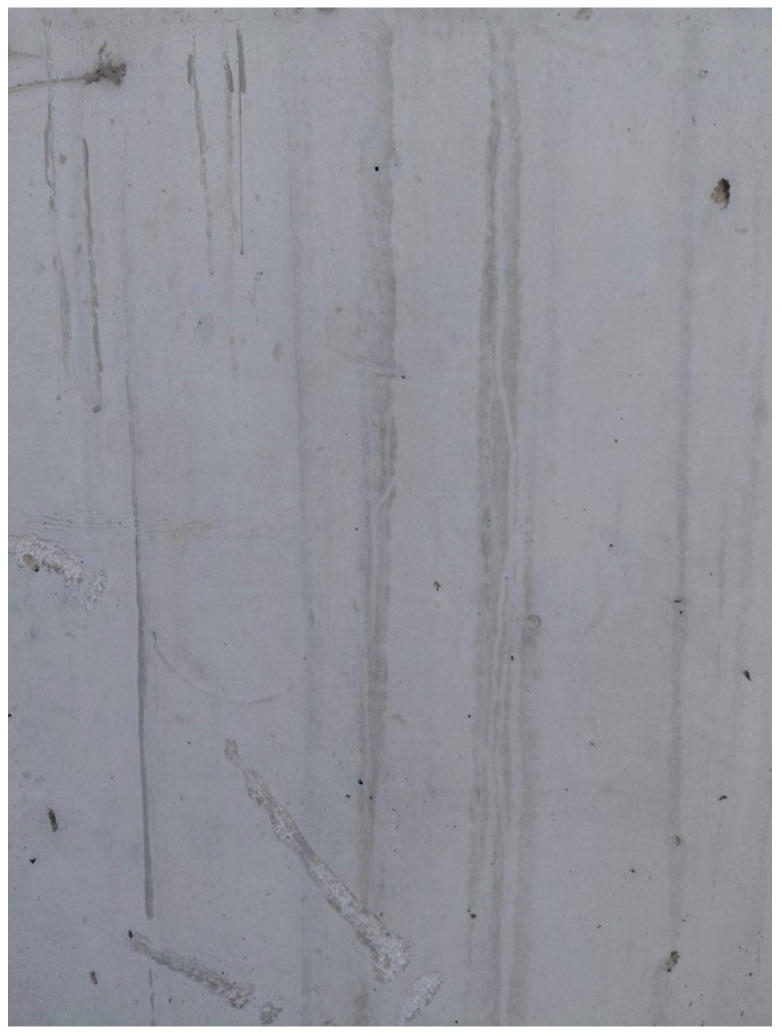
Surface roughness of the test surface after 3 min of application.

**Figure 17:**
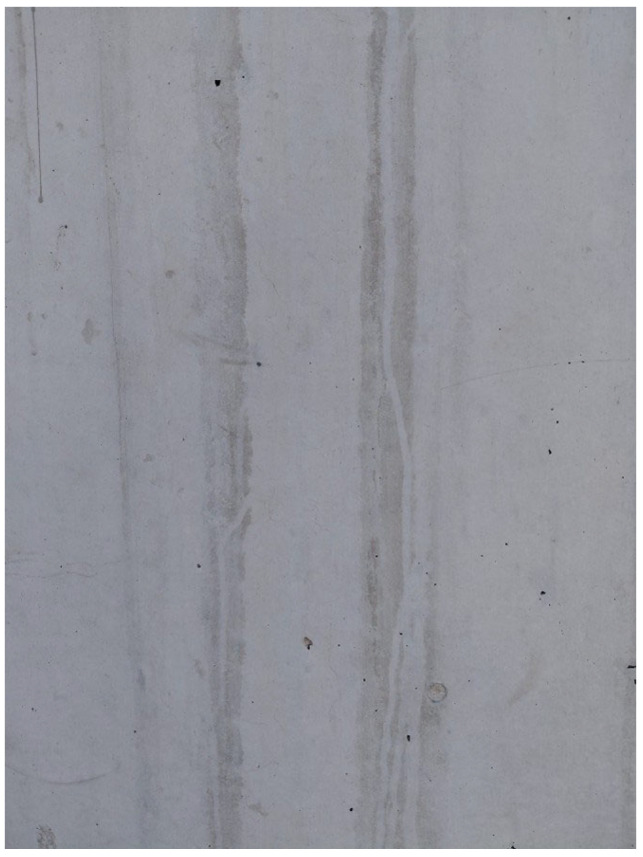
Surface roughness of the test surface after 6 min of application.

In addition to the in-mold PZT excitation, a portable proof-of-concept prototype was developed to improve surface quality under field conditions. The prototype was designed based on the symmetric placement of different numbers of PZT transducers (2, 4, and 6) on a steel plate. The objective was to reduce surface roughness using the vibration energy generated by simultaneously activated transducers. The plate was applied in contact with an early-age (fresh-to-initial-setting stage) concrete surface and excited for 1 min at a total nominal driver output power of 240 W (4 × 60 W), and a qualitative leveling effect was observed on the surface. This prototype constitutes a feasibility-oriented proof-of-concept aimed at translating the image processing-based reduction in surface roughness reported in this study (from 29.8% to 4.3%) into a practical application scenario. In this context, prototype trials were conducted on a limited basis and relied on qualitative assessment to demonstrate feasibility. The prototype surface-leveling setup used in the application scenario is shown in Figure 15. The surface image after 3 min of application is shown in Figure 16, whereas the surface image after 6 min of application is presented in Figure 17.

**Figure 18:**
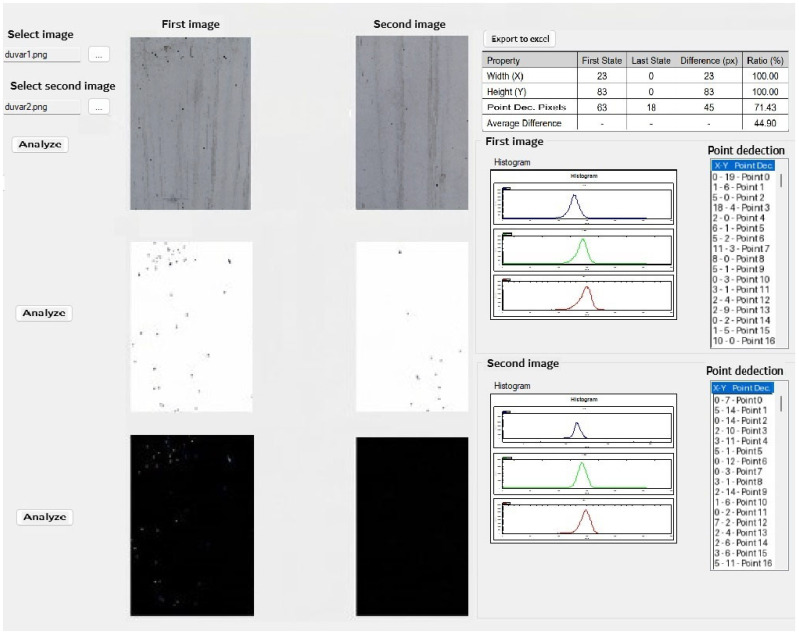
Analysis program interface output for hole/void detection on the surfaces before and after PZT application.

Additionally, an image processing-based analysis program was developed to quantitatively assess the differences in surface condition before and after the application. Based on the evaluation performed using the developed program, the number of detected holes/voids was 81 on the untreated surface (without PZT), while this value decreased to 18 on the surface treated with the PZT system. This finding indicates an approximately 77.8% reduction in the number of holes/voids. The program interface output of the fill-void analysis is presented in Figure 18.

Surface roughness issues encountered in field applications can be substantially mitigated using PZT transducers. When combined with frequencies optimized for the target surface material, PZT-based actuation may represent a cost-effective option for significantly reducing surface roughness. may have potential for practical surface applications, although further validation is required. In addition, features such as portability and low weight indicate that such systems could be well suited for practical surface applications. Moreover, transducers placed symmetrically on a plate of a given size may transfer the generated transverse and longitudinal waves to the target surface more efficiently, offering an inspiring approach for alleviating surface roughness without adversely affecting strength. In this context, PZT transducers mounted on a plate can be applied to the target surface for prescribed durations with the aim of surface leveling. The experimental findings obtained in the present study are promising for further research. In addition to achieving a pronounced reduction in surface roughness, the approach has the potential to substantially reduce cost compared with related studies in this area. Its portable nature may also enable applicability across a wide range of surfaces. The proposed plate equipped with a PZT array can be tested on different concrete specimens, and based on the resulting dataset, artificial intelligence algorithms may be employed to determine the most optimized excitation frequency and duration for a given material, thereby markedly enhancing usability.

Overall, the proposed PZT vibration system does not constitute a direct alternative to conventional compaction methods but provides selective advantages in improving surface quality and facilitating the removal of entrapped air. The reduction in compressive strength under prolonged high-frequency excitation can be attributed to microstructural disruption mechanisms. Unlike conventional low-frequency vibration, high-frequency oscillations may induce localized stress concentrations, leading to microcrack initiation within the cement matrix. In addition, particle migration and segregation effects may occur due to resonance-induced movement of aggregates, resulting in non-uniform density distribution.

Furthermore, owing to its low power consumption, it may reduce costs—particularly for surface applications—while improving practicality by alleviating implementation challenges. However, optimizing vibration parameters is critical to avoid adverse microstructural effects. This study highlights the complex interactions among compaction method, aggregate fraction, specimen density, and mechanical properties, and underscores the need for optimized vibration protocols in concrete processing. In future work, the effects of different excitation frequencies and shorter application durations could be investigated to minimize microcrack formation. The use of admixtures may also be considered to enhance matrix integrity and compensate for potential strength losses.

Recent advancements have also explored the use of hybrid evaluation techniques combining ultrasonic testing with digital imaging to improve the reliability of concrete quality assessment. Studies have demonstrated that integrating multiple measurement approaches provides a more comprehensive understanding of internal defects and surface conditions, particularly in heterogeneous materials such as concrete [28,29,30].

Moreover, recent investigations into ultrasonic and high-frequency excitation systems have shown that vibration parameters such as frequency, duration, and power significantly influence both microstructure development and mechanical performance. While some studies report improvements in density and surface uniformity, others highlight the risk of microcracking and strength reduction under excessive or prolonged excitation [8,31].

This study introduces a high-frequency (40 kHz), low-power PZT-based vibration protocol integrated into concrete molds as an alternative to conventional low-frequency, high-power vibration practices. The work provides a comparative dataset (mass, UPV, compressive strength) across four aggregate fractions under controlled excitation durations (0, rodding/tamping, 1 min, 3 min). Surface quality improvement is quantified via image processing metrics (Light Map 150, Specular Map 150), demonstrating a substantial reduction in rough-area fraction (≈29.8% → ≈4.3%). A portable, proof-of-concept PZT plate prototype is proposed for practical surface leveling, and preliminary trials indicate a marked reduction in detected surface voids.

A portable proof-of-concept PZT plate system was developed to explore potential practical applications. Preliminary observations indicated improved surface leveling and reduced visible defects. However, further large-scale validation is required.

The main contribution of this study lies not in demonstrating performance improvement but in revealing the limitations, parameter sensitivity, and application boundaries of high-frequency piezoelectric vibration in concrete processing.

## Figures and Tables

**Table 1:** UPV test data for hardened concrete.

Specimen ID	Specimen Name	UPV Result (m/s)
1	Fine Aggregate-Untreated	3640
2	Fine Aggregate-Rodded & Tamped	3640
3	Fine Aggregate-Vibrated (1 min)	3667
4	Fine Aggregate-Vibrated (3 min)	3605
5	Medium Aggregate-Untreated	3614
6	Medium Aggregate-Rodded & Tamped	3092
7	Medium Aggregate-Vibrated (1 min)	3113
8	Medium Aggregate-Vibrated (3 min)	3107
9	Medium-Coarse Aggregate-Untreated	3826
10	Medium-Coarse Aggregate-Rodded & Tamped	3605
11	Medium-Coarse Aggregate-Vibrated (1 min)	3787
12	Medium-Coarse Aggregate-Vibrated (3 min)	3296
13	Coarse Aggregate-Untreated	4010
14	Coarse Aggregate-Rodded & Tamped	4021
15	Coarse Aggregate-Vibrated (1 min)	3554
16	Coarse Aggregate-Vibrated (3 min)	4143

**Table 2:** Mass data for hardened concrete.

Specimen ID	Specimen Name	Mass (kg)	Average Mass (kg)
1	Fine Aggregate-Untreated	7.04	6.69
2	Fine Aggregate-Rodded & Tamped	6.99	
3	Fine Aggregate-Vibrated (1 min)	6.48	
4	Fine Aggregate-Vibrated (3 min)	6.26	
5	Medium Aggregate-Untreated	7.12	6.82
6	Medium Aggregate-Rodded & Tamped	7.13	
7	Medium Aggregate-Vibrated (1 min)	6.53	
8	Medium Aggregate-Vibrated (3 min)	6.56	
9	Medium-Coarse Aggregate-Untreated	6.83	6.57
10	Medium-Coarse Aggregate-Rodded & Tamped	6.84	
11	Medium-Coarse Aggregate-Vibrated (1 min)	6.28	
12	Medium-Coarse Aggregate-Vibrated (3 min)	6.31	
13	Coarse Aggregate-Untreated	7.04	6.82
14	Coarse Aggregate-Rodded & Tamped	6.96	
15	Coarse Aggregate-Vibrated (1 min)	6.63	
16	Coarse Aggregate-Vibrated (3 min)	6.65	

**Table 3:** Compressive strength data for hardened concrete.

Specimen ID	Specimen Name	Destructive Test Duration (s)	Compressive Strength (MPa)	Average Compressive Strength (MPa)
1	Fine Aggregate-Untreated	41.43	20.62	14.17
2	Fine Aggregate-Rodded & Tamped	44.80	19.96	
3	Fine Aggregate-Vibrated (1 min)	104.90	11.37	
4	Fine Aggregate-Vibrated (3 min)	36.51	4.74	
5	Medium Aggregate-Untreated	45.67	22.73	13.49
6	Medium Aggregate-Rodded & Tamped	43.31	19.75	
7	Medium Aggregate-Vibrated (1 min)	26.19	5.20	
8	Medium Aggregate-Vibrated (3 min)	47.81	6.27	
9	Medium-Coarse Aggregate-Untreated	38.88	16.25	10.22
10	Medium-Coarse Aggregate-Rodded & Tamped	43.10	16.43	
11	Medium-Coarse Aggregate-Vibrated (1 min)	17.11	3.91	
12	Medium-Coarse Aggregate-Vibrated (3 min)	36.52	4.28	
13	Coarse Aggregate-Untreated	43.48	19.25	14.50
14	Coarse Aggregate-Rodded & Tamped	37.67	17.97	
15	Coarse Aggregate-Vibrated (1 min)	143.39	10.64	
16	Coarse Aggregate-Vibrated (3 min)	106.53	10.14	

## Data Availability

The original contributions presented in this study are included in the article. Further inquiries can be directed to the corresponding author.
